# Functional characterization of Vip3Ab1 and Vip3Bc1: Two novel insecticidal proteins with differential activity against lepidopteran pests

**DOI:** 10.1038/s41598-017-11702-2

**Published:** 2017-09-11

**Authors:** Marc D. Zack, Megan S. Sopko, Meghan L. Frey, Xiujuan Wang, Sek Yee Tan, Jennifer M. Arruda, Ted T. Letherer, Kenneth E. Narva

**Affiliations:** Dow AgroSciences, 9330 Zionsville Road, Indianapolis, IN 46268 USA

## Abstract

In this work, we characterized 2 novel insecticidal proteins; Vip3Ab1 and Vip3Bc1. These proteins display unique insecticidal spectra and have differential rates of processing by lepidopteran digestive enzymes. Furthermore, we have found that both proteins exist as tetramers in their native state before and after proteolysis. In addition, we expressed truncated forms and protein chimeras to gain a deeper understanding of toxin specificity and stability. Our study confirms a role for the C-terminal 65 kDa domain in directing insect specificity. Importantly, these data also indicate a specific interaction between the 20 kDa amino terminus and 65 kDa carboxy terminus, after proteolytic processing. We demonstrate the C-terminal 65 kDa to be labile in native proteolytic conditions in absence of the 20 kDa N-terminus. Thus, the 20 kDa fragment functions to provide stability to the C-terminal domain, which is necessary for lethal toxicity against lepidopteran insects.

## Introduction

*Bacillus thuringiensis* (Bt) is the bacterial source of insecticidal proteins that have been expressed in genetically modified crops to confer resistance to pest feeding damage and crop loss. Many insecticidal proteins are produced as crystalline inclusion bodies during the late log-growth to sporulation phase of bacterial growth. Insecticidal crystals are then solubilized in the digestive tract of insects where they are activated by midgut proteases to form active toxins, which disrupt membrane integrity and cause midgut epithelial cell lysis through specific interaction with cellular receptors^1, 2^. Another class of Bt toxins are produced during the vegetative growth phase and are termed Vegetative insecticidal proteins (Vip’s,^3^). The Vip proteins are composed of 4 subgroups; Vip1, Vip2, Vip3 and Vip4. Vips 1 and 2 are frequently active against coleopteran species of insects and function as binary insecticidal proteins utilizing ADP-ribosyltransferase activity to exert toxic effects on target insect cells^2, 4–6^. Vip3 proteins have activity against a broad spectrum of economically important lepidopteran insects such as fall armyworm (*Spodoptera frugiperda*), beet armyworm (*Spodoptera exigua*), tobacco budworm (*Heliothis virescens*), corn earworm (*Helicoverpa zea*), and marginal activity against European corn borer (*Ostrinia nubilalis*)^3, 7–9^. The Vip4 subgroup is composed of only one member, Vip4Aa1, with no known insecticidal activity^5^.

Because of their broad spectrum of insecticidal activity, Vip3 proteins have been used for the development of transgenic crops such as event COT102 cotton and event MIR162 corn^10^. Thus, the Vip3 subfamily is amenable to transgenic plant protection; however, much less is understood about Vip3 mode of action. The proposed sequence of biochemical events leading to toxic effect is similar to those described for classic crystalline Bt three domain toxins^2^. In the current proposed mode of action model, Vip3 proteins are first ingested and then activated by proteolytic removal of a ~20 kDa N-terminal pro-domain. Next, the ~65 kDa active toxin “core” is proposed to bind specific receptors in the insect midgut epithelium resulting in cell lysis and loss of membrane integrity^11–14^. However, there is little direct biochemical evidence to substantiate this sequence of events and the majority of research has been performed on a single subclass of Vip3A proteins that share greater than 95% amino acid sequence identity. A more detailed understanding of the Vip3 mechanism of action, activation, and specificity is needed to support further development of new Vip3 insecticidal proteins as insect resistance traits.

In this work, we have functionally characterized ~20 kDa and ~65 kDa Vip3 domain interactions using two new members of the Vip3 family, Vip3Ab1 and Vip3Bc1. These insecticidal proteins demonstrated clear differences in proteolytic processing that did not correlate with activity against susceptible insects. In addition, we observed that both proteins exist as tetramers prior to stepwise enzymatic processing by midgut enzymes. Interestingly, Vip3Ab1 and Vip3Bc1 proteins persist as tetramers in solution after enzymatic processing, indicating a direct and sustained interaction between the products of partial proteolysis. We have also expressed and purified the C-terminal portions of Vip3Ab1 and Vip3Bc1, often referred to as the toxic “core”^5^, and observed no insecticidal activity for either protein. Lastly, we have synthesized chimeric Vip3 proteins to demonstrate that the C-terminal ~65 kDa domain is responsible for toxin specificity and that the ~20 kDa N-terminal maintains proteolytic stability and is required for toxicity. To our knowledge, this is the first demonstration that Vip3 insecticidal proteins require interaction between amino- and carboxy- termini for toxicity. Despite highly conserved sequence identity of the amino terminal regions, our data indicate that interactions of the amino terminal regions with the carboxy terminus promote oligomerization and provide proteolytic stability required for lethal toxicity. Thus, we have demonstrated that the Vip3 family of proteins undergo a unique processing and activation pathway that is different than the 3 domain Cry family of Bt proteins. These new data shed new light on Vip3 domain functionality, indicating that Vip3 proteins may have additional potential for the control of commercially important lepidopteran pests.

## Results

### Comparison of Vip3Ab1,Vip3Bc1 and Vip3 chimeras

**Figure 1:**
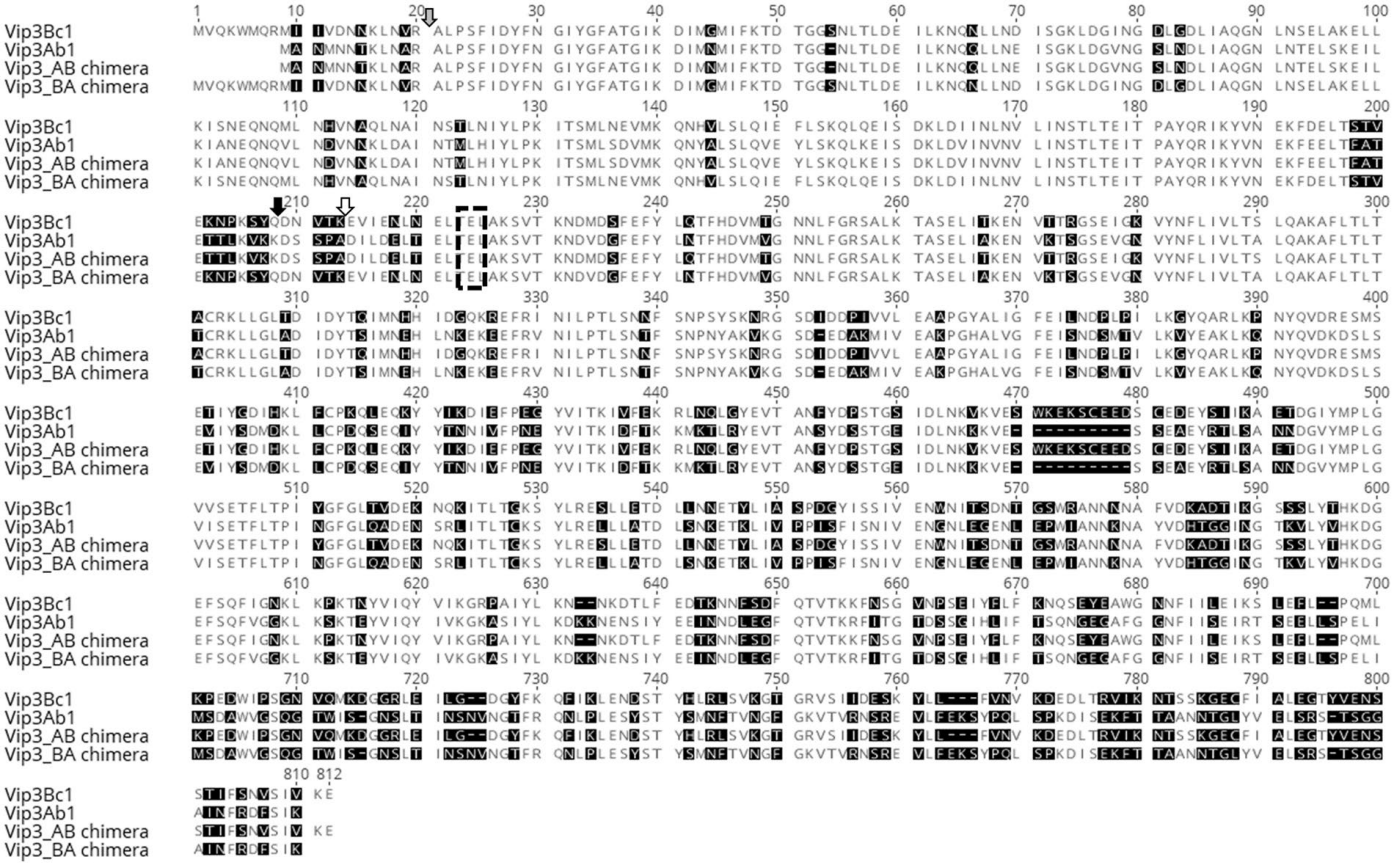
Sequence alignment of Vip3 proteins in this study. The top line is Vip3Bc1 used the reference sequence for all amino acid numbering. Vip3Ab1, Vip3_AB, and Vip3_BA chimeras are also listed. Black background shading is used to highlight amino acid diversity between proteins (BLOSUM62 substitution matrix). The grey shaded arrow highlights the initial protease cleavage site common to all Vip3 proteins. The white arrow denotes the Vip3Bc1 gut protease site and the black arrow denotes the Vib3Ab1 gut protease site identified by N-terminal sequencing. A dashed line box marks the site at which Vip3_AB and Vip3_BA chimeras were generated.

**Figure 2:**
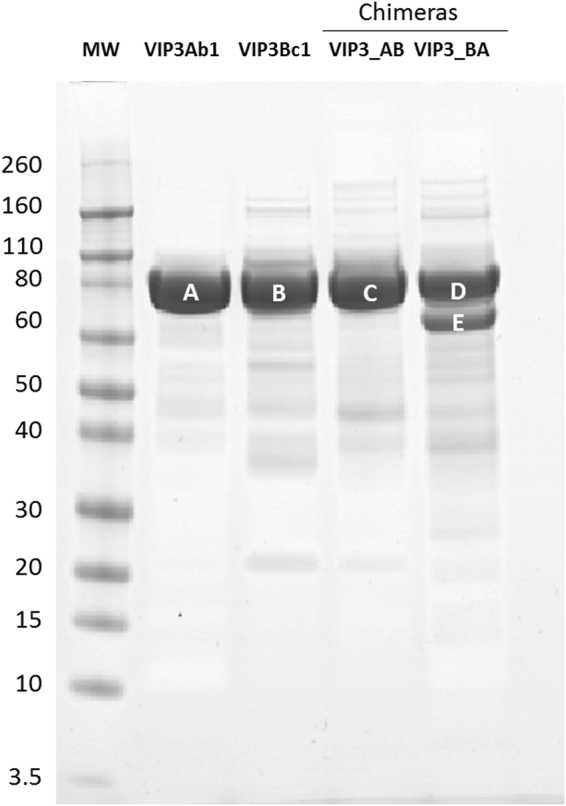
SDS-PAGE analysis of Vip3 proteins expressed and purified from Pseudomonas. The N-terminal sequence of bands A and C was ^10^ANMNN (amino acid #2 by Vip3Ab1 numbering) and for bands B and D was ^2^VQKWM. Vip3_BA preparations also contained a partially processed form of the protein with an N-terminus of ^190^NEKFD (**E**).

An amino acid sequence alignment of Vip3Ab1 and Vip3Bc1 is illustrated in Fig. 1. By conventional terminology, members of the Vip3 class are at least 45% identical and a shared secondary ranking (A, B, etc.) indicates that two Bt proteins are at least 78% identical across the length of the protein^15, 16^. Vip3Bc1 has 61% amino acid identity to Vip3Ab1. The Vip3Bc1 N-terminal sequence is unique as the N-terminal methionine is 8 amino acids upstream of the more conserved sequence, MANMNNTKLN, found in Vip3Ab1 and other Vip3 family members^3, 17^. Vip3Bc1 also has a short insertion of ^472^KEKSCEEDSCEDE^484^, which is similar to a longer repeat of charged amino acids found in Vip3Ba1 (^472^KEDCCEEDCCEEDCCEEDCCEE^493^)^18^. In total, Vip3Bc1 is comprised of an additional 26 amino acids relative to Vip3Ab1 and will used as the reference sequence to for amino acid location of both proteins. Both proteins expressed with only the first methionine removed and observed molecular weight consistent with the predicted mass of ~85 kDa (Fig. 2). Vip3Ab1 contains a canonical serine proteinase cleavage motif at ^205^ **KVKK**↓DSSP, whereas Vip3Bc1 contains 3 substitutions at this site resulting in ^205^ **KSYQ**↓DNVT. Both genes expressed soluble proteins in *Pseudomonas fluorescens* and had insecticidal activity against *Pseudoplusia includens* (Table 1). Vip3Ab1 had lethal activity on *H. zea* and *S. frugiperda* whereas Vip3Bc1 had lethal activity against *O. nubilalis*.

**Table 1:** Insect bioassay results.

	Insect Target	LC_50_ (ng/cm^2^)	Lower 95% CI	Upper 95% CI
Vip3Ab1	*Helicoverpa zea*	555.7	338.4	936.6
	*Spodoptera frugiperda*	72.6	49.4	120.6
	*Ostrinia nubilalis*	>9000	NC	NC
	*Pseudoplusia includens*	124.2	73.7	199.1
Vip3Bc1	*Helicoverpa zea*	>9000	NC	NC
	*Spodoptera frugiperda*	>9000	NC	NC
	*Ostrinia nubilalis*	485.3	298.1	787.6
	*Pseudoplusia includens*	1004.2	580.2	1915.6
Vip3_AB	*Helicoverpa zea*	>9000	NC	NC
	*Spodoptera frugiperda*	>9000	NC	NC
	*Ostrinia nubilalis*	>9000	NC	NC
	*Pseudoplusia includens*	>9000	NC	NC
Vip3_BA	*Helicoverpa zea*	>9000**	NC	NC
	*Spodoptera frugiperda*	>9000**	NC	NC
	*Ostrinia nubilalis*	>9000	NC	NC
	*Pseudoplusia includens*	>9000**	NC	NC

LC50 concentration was calculated using a Probit analysis of the sum of dead and moribund insects relative to the total number of treated insects. “**”Indicates growth inhibition observed on treated insects at multiple concentrations. “NC” indicates that confidence intervals (CI) were not calculated.

**Figure 3:**
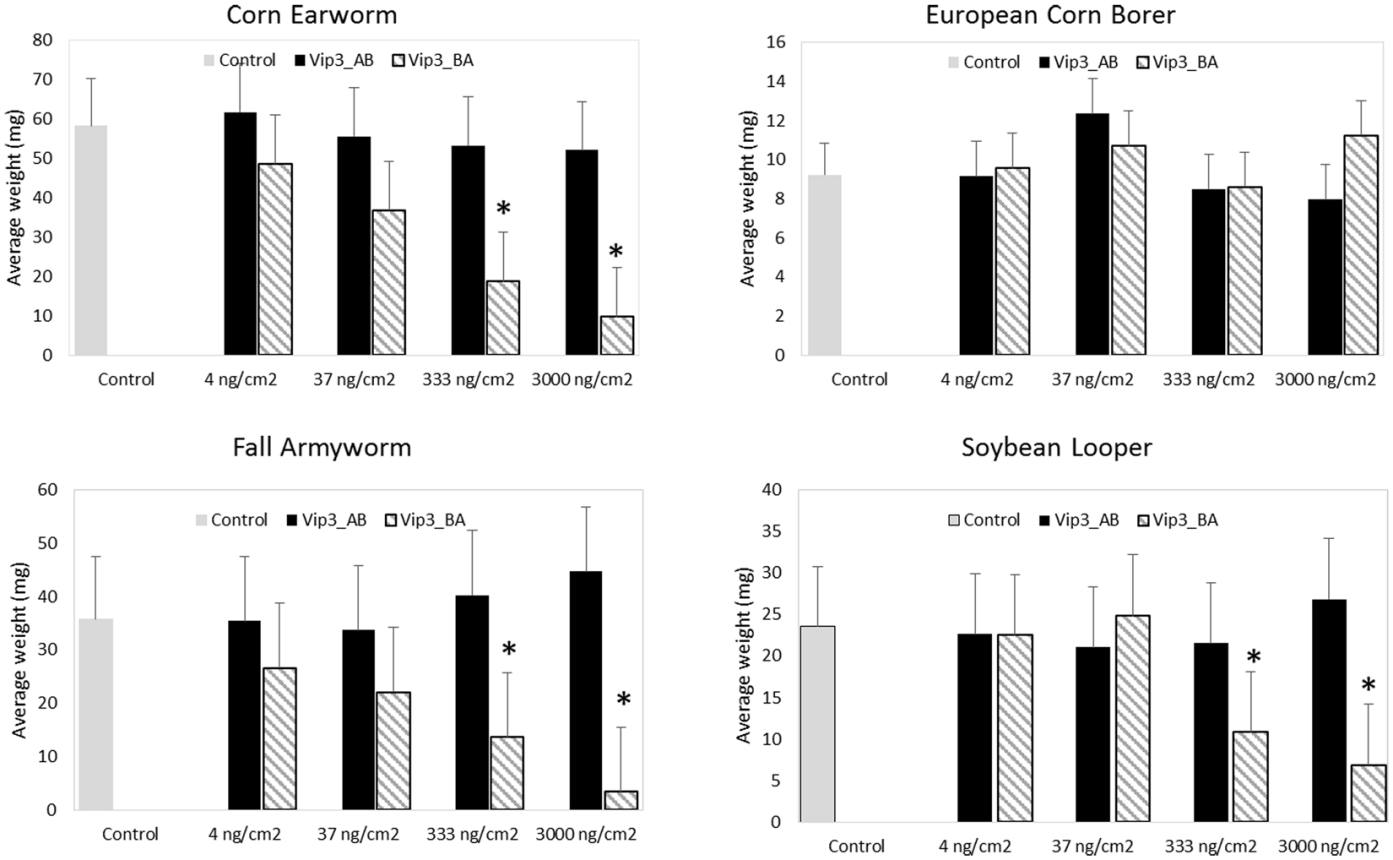
Effects of Vip3_AB and Vip3_BA chimeras on insect weight after 5 days of exposure on diet overlay bioassay. Bars represent the average mass of 16 insects per treatment with standard error of the mean. Statistical analysis was performed as described in Materials and Methods for comparing insect weight under Vip3_AB or Bip3_BA chimera treatments to that of untreated controls. Asterisks indicate a significant (P < 0.05) decrease in mass relative to untreated controls.

To investigate the role of processing and regions of diversity from Vip3Ab1 and Vip3Bc1, chimeric proteins Vip3_AB (N-terminal domain of Vip3Ab1 and C-terminal domain of Vip3Bc1) and Vip3_BA (N-terminal domain of Vip3Bc1 and C-terminal domain of Vip3Ab1) were expressed and purified from *P. fluorescens*. Glutamic acid at position #224 (^224^ELAKS) was chosen as the site to generate chimeras because this was within a short sequence conserved between Vip3Ab1 and Vip3Bc1 from amino acids 221–233 (based on Vip3Bc1 numbering, Fig. 1). Sequence exchange at this site avoided disruption of upstream processing sites, which made these proteins ideal for investigation of domain function and processing. Both toxin chimeras expressed as full length proteins with the N-terminal methionine removed. However, Vip3_BA was also present as a partially processed protein with the N-terminus at ^190^NEKFD (Fig. 2). Neither chimera produced lethal or morbid effects against the 4 insects utilized in this study. However, Vip3_BA-treated *H. zea, P. includens, and S. frugiperda* were smaller in size and showed strong inhibition of growth relative to buffer-treated insects (Fig. 3) whereas Vip3_AB showed no toxic effects on any of the tested insects.

### Processing of Vip3 proteins by lepidopteran gut enzymes

**Figure 4:**
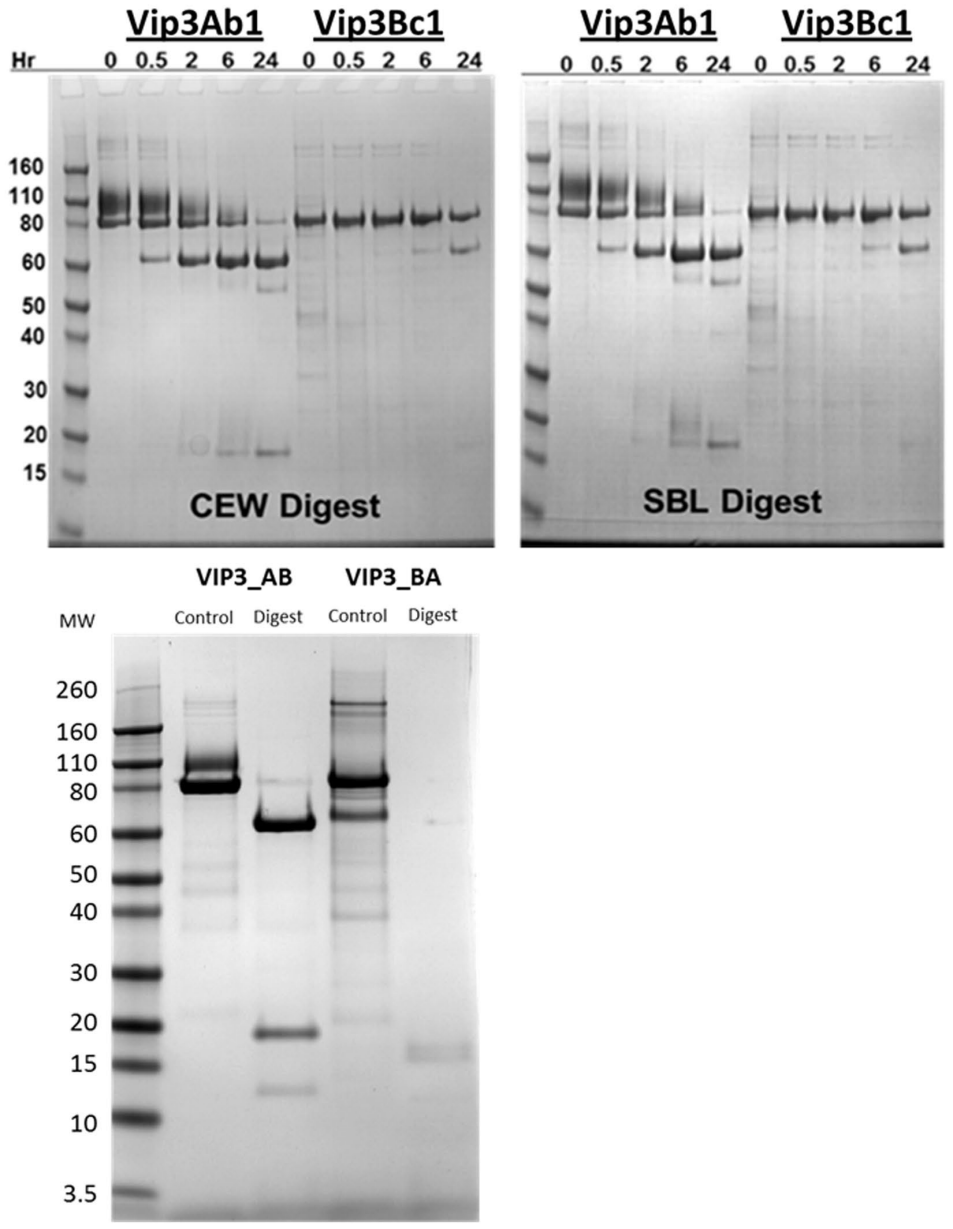
Top Panel: SDS-PAGE analysis of time course digestion of Vip3Ab1 and Vip3Bc1 with *H. zea* and *P. includens* gut enzymes. Vip3Ab1 and Vip3Bc1 proteins (150 µg/mL) were incubated with gut fluids from *H. zea* (left) and *P. includens* (right) at 30 °C for various time intervals at pH 10.0. Bottom Panel: SDS-PAGE analysis of overnight digestion of Vip3 chimeras with *H. zea* gut enzymes. Vip3_AB and Vip3_BA proteins (110 µg/ml) were incubated with *H. zea* gut fluids for 16 hours at 30 °C in a total volume of 100 µL at pH 10.0. All reactions were stopped with protease inhibitors and 30 µL of the reaction loaded as described in Materials and Methods. Equivalent lanes were loaded and blotted onto a PVDF membrane for N-terminal sequencing.

We analyzed the time course of *H. zea* and *P. includens* midgut proteolytic processing of Vip3Ab1 and Vip3Bc1 proteins by SDS-PAGE (Fig. 4, top panel). All digestion experiments were performed at pH 10.0 as this pH is similar to the alkaline midgut environment of lepidoptera. In this condition, we observed the formation of an additional Vip3Ab1 band at ~110 kDa that was not apparent in gels using protein in pH 8.0 buffer. This band and the full length ~85 kDa Vip3Ab1 behaved similar during the timecourse of digestion experiments. Both Vip3Ab1 and Vip3Bc1 were processed to ~65 kDa and ~20 kDa products. However, Vip3Bc1 was processed at a much slower rate than Vip3Ab1 regardless of the source of lepidopteran midgut enzymes. Vip3Ab1 was processed at ^209^Asp as expected based on the location of the ^205^KVKK↓D cleavage motif. Analysis of earlier time points (<6 hr) indicated the larger molecular weight protein at ~85 kDa was processed at ^21^Ala. This was also the N-terminus of the lower molecular weight product at ~20 kDa. Taken together, this suggests stepwise proteolysis where a small amount of the N-terminus is removed prior to further processing. Vip3Bc1, which lacks a conserved dibasic cleavage site, was processed at ^214^Glu. Similarly to Vip3Ab1, N-terminal sequencing of the ~85 kDa protein at early time points indicated that a small portion of the N-terminus was removed at ^21^Ala. Next, chimeric Vip3 proteins were analyzed after incubation overnight with *H. zea* midgut extracts (Fig. 4, bottom panel). SDS-PAGE analysis showed the production of similar molecular weight products with Vip3_AB, which was processed at ^21^Ala and ^209^Asp as anticipated (Fig. 4, bottom panel). However, Vip3_BA was almost completely degraded to low molecular weight products of <10 kDa.

### Size exclusion chromatographic analysis of Vip3 toxins

**Figure 5:**
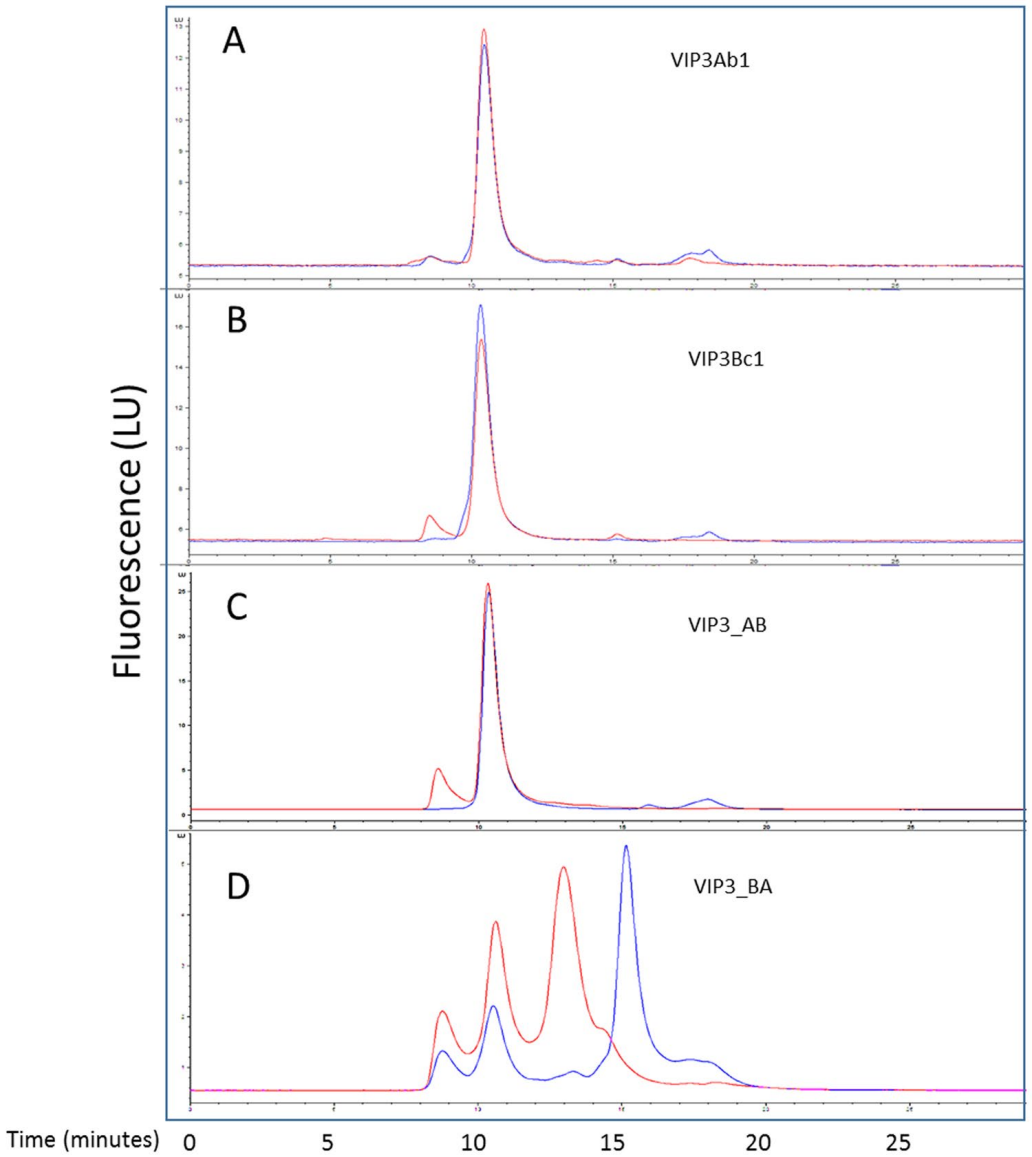
Size exclusion chromatography of Vip3 proteins. Both Vip3Ab1 (panel A) and Vip3Bc1 (panel B) had a retention time of ~10.13 min before (Red) and after (Blue) *H. zea* gut enzyme digestion that was consistent with a tetrameric state based on molecular weight standards. Vip3_AB (panel C) had a retention time of ~10.13 min before (Red) and after (Blue) *H. zea* gut enzyme digestion. However, Vip3_BA (panel D) had multiple peaks consistent with aggregated (~8.8 min), tetrameric (~10.1 min) and monomeric (~13.1 min) species prior to *H. zea* gut enzyme digestion (Red). Monomeric forms were digested more rapidly than tetrameric forms throughout the 24 hour digestion (Blue).

**Figure 6:**
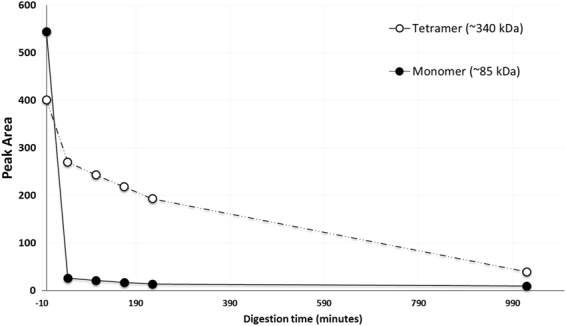
Time course of Vip3_BA digestion by *H. zea* gut enzymes. Vip3_BA was incubated with *H. zea* gut enzymes at 23 °C and injected onto an analytical SEC column after different time intervals. Protein peak areas at 10.1 minutes and 13.1 minutes, consistent with monomeric and tetrameric species of protein, were compared. The monomeric protein peak was nearly completely degraded after 45 minutes (solid line, closed circles) whereas tetrameric species (dotted line, open circles) were stable for a longer period of time.

We next investigated the existence of Vip3 oligomers by analytical size exclusion chromatography (SEC; Fig. 5 panels A and B). Vip3Ab1 and Vip3Bc1 both migrated with a retention time consistent with a tetramer (10.1 min, ~340 kDa) and a minor peak found in the void volume, indicating that the majority of Vip3Ab1 and Vip3Bc1 preparations were tetrameric while in solution. The Vip3_AB chimera also appeared tetrameric in native conditions (Fig. 5, panel C). However, the counterpart Vip3_BA chimera was approximately 50% monomeric and 50% tetrameric when analyzed by SEC (Fig. 5, panel D). With exception of the Vip3_BA chimera, the retention time consistent with a tetramerization persisted even after overnight processing with *H. zea* midgut enzymes. Collected fractions confirmed processing of Vip3 proteins (not shown). Interestingly, time course SEC analysis of Vip3_BA indicated that monomeric forms of this protein were completely processed to lower molecular weight products (<30 kDa) within 45 minutes as estimated by retention time (Fig. 6). However, the tetrameric portion of the Vip3_BA chimera was more resistant to degradation by midgut proteases which required 17 hours for complete degradation (Fig. 6).

### Analysis of expressed C-terminal ~65 kDa domains of Vip3Ab1 and Vip3Bc1

**Figure 7:**
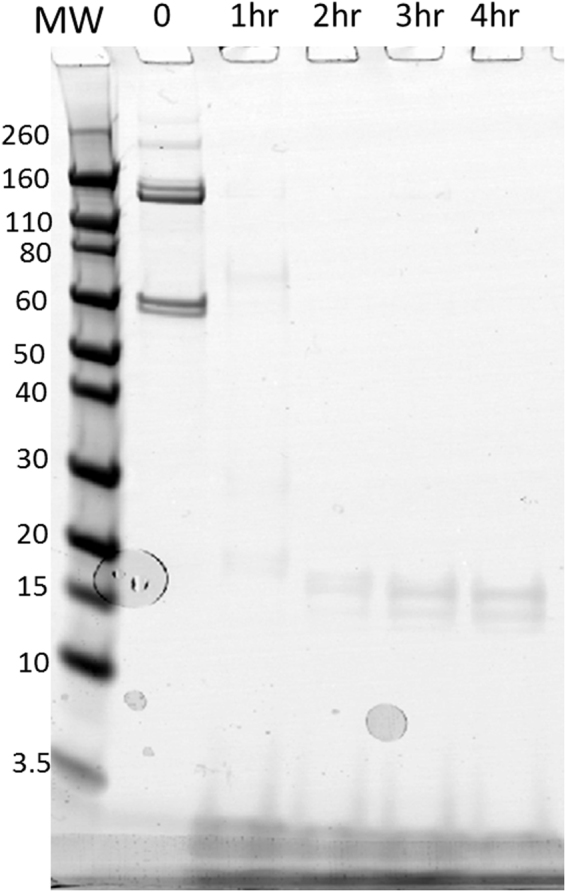
Time course digestion of Vip3Ab1 C-terminal ~65 kDa domain with *H. zea* gut enzymes. Proteins (150 µg/ml) was incubated with *H. zea* gut fluids for indicated times at 30 °C in a total volume of 100 µL at pH 10.0. The reaction was stopped with protease inhibitors and 30 µL of the reaction was analyzed by SDS-PAGE as described in Materials and Methods. Similar results were observed with the C-terminal region of Vip3Bc1 (data not shown).

Lastly, we characterized the stability and toxicity of the C-terminal region of Vip3Ab1 and Vip3Bc1. Each protein, beginning at Leu^222^ (numbering based on Vip3Bc1 sequence) was expressed and purified from recombinant *P. fluorescens* strains. When analyzed by SEC, both proteins eluted as dimers with an estimated molecular weight of ~120 kDa. However, the C-terminus of Vip3Bc1 resolved as a single band at ~60 kDa by SDS-PAGE analysis. Interestingly, the Vip3Ab1 C-terminal protein migrated as two different species with molecular weights of 120 kDa and 60 kDa (Fig. 7). Both proteins were rapidly degraded by CEW gut enzymes and neither demonstrated growth inhibition or lethality against susceptible lepidopteran pests.

## Discussion

Vip3 insecticidal proteins are attractive for the development of transgenic insect-resistant crops owing to their potent and broad spectrum activity against lepidopteran pests. Extant data indicates that Vip3 proteins do not bind to the same receptors as typical crystalline three-domain Bt proteins expressed in transgenic crops^19–21^. However, information on structure and function of the Vip3 proteins is lacking. Our work has focused on providing fundamental information on the functional domains of a Vip3Ab1, as well as Vip3Bc1, a novel representative of the less characterized Vip3B subfamily.

### Vip3Ab1 and Vip3Bc1 have different spectra of activity against insect pests

Vip3Ab1 and Vip3Bc1 are 75% identical from amino acids 1-222 and 54% identical from amino acids 223–812. As noted by other groups, the majority of Vip3 diversity is found at the C-terminus, which has been suggested to be critical for insecticidal activity and specificity^5, 22^. Vip3Ab1 has lethal activity on *H. zea, S. frugiperda*, and *P. includens*. This is not surprising, as several members of the Vip3A family have potent activity within the *Spodoptera* or *Helicoverpa* genus of insects^7, 12, 14, 22, 23^. Also in agreement with previous Vip3A work, Vip3Ab1 lacks insecticidal activity against *O. nubilalis*. In this study, we show that Vip3Bc1 has insecticidal activity against *O. nubilalis* and no activity on *H. zea* or *S. frugiperda*. Rang *et al*.^18^ observed growth inhibition of *O. nubilalis* by Vip3Ba1, although, no mortality was observed. Therefore, Vip3Bc1 represents the second member of the Vip3B subfamily with insecticidal activity against *O*. *nubilalis*. Thus, Vip3Ab1 and Vip3Bc1 have differential insecticidal spectra and are good candidates to evaluate structure –function relationships compared to Vip3A proteins.

### Vip3Bc1 is less prone to enzymatic processing than Vip3Ab1

Yu *et al*.^14^ have shown that Vip3Aa is readily cleaved by midgut proteases from both susceptible and non-susceptible insects and so concluded that toxicity was determined by insect-specific receptor binding. Lee *et al*. followed on this hypothesis and employed an intact midgut voltage clamp assay to show that only processed Vip3A resulted in pore formation in midguts from susceptible, but not non-susceptible, insects^12^. Taken together, the studies indicate a minimal requirement for processing, but still support the requirement to bind to a specific receptor. Therefore, we investigated Vip3 processing using midgut enzymes from *H. zea* and *P. includens*. Enzymatic digests were carried out at pH 10 to mimic the alkaline pH of the lepidopteran gut. In these conditions, Vip3Bc1 was processed at a much slower rate than Vip3Ab1 by *H. zea* and *P. includens* gut enzymes. This is an intriguing finding as *P. includens* is susceptible to Vip3Bc1 despite relatively slow midgut protease processing. The slow rate of digestion may be due to the lack of a canonical serine protease cleavage site. Based on these data, we cannot conclude if processing is an absolute requirement for toxicity by Vip3B family members. However, it is possible that membrane-bound proteinases not present in our soluble gut enzyme preparations are responsible for processing of Vip3B toxins *in vivo*.

### Processing of Vip3Ab1 and Vip3Bc1 undergo similar stepwise proteolysis

In this study, we observed an early (~30 min) initial cleavage event that occurred with both Vip3Ab1 and Vip3Bc1 at ^13^ALPSF prior to subsequent downstream cleavage events. This first step in processing removes the signal peptide from the N-terminus. It has been noted that Vip3 proteins are secreted by native Bt cells with putative N-terminal secretion peptides intact^3^. Furthermore, Selvapandiyan *et al*.^24^ reported that removal of the N-terminal 39 amino acids from Vip3Aa9 has no effect on toxicity towards *Chilo partellus* but had markedly reduced activity towards *Spodoptera litura*. Other research indicates that N-terminal extension of Vip3 proteins affects the rate of processing and insect activity^25^. As the ^21^ALPSF site is well-conserved amongst all current subfamilies of Vip3 proteins, this primary processing step may be an important step common to Vip3A and Vip3B subfamilies that could be involved in maintaining a stable active conformation.

**Figure 8:**
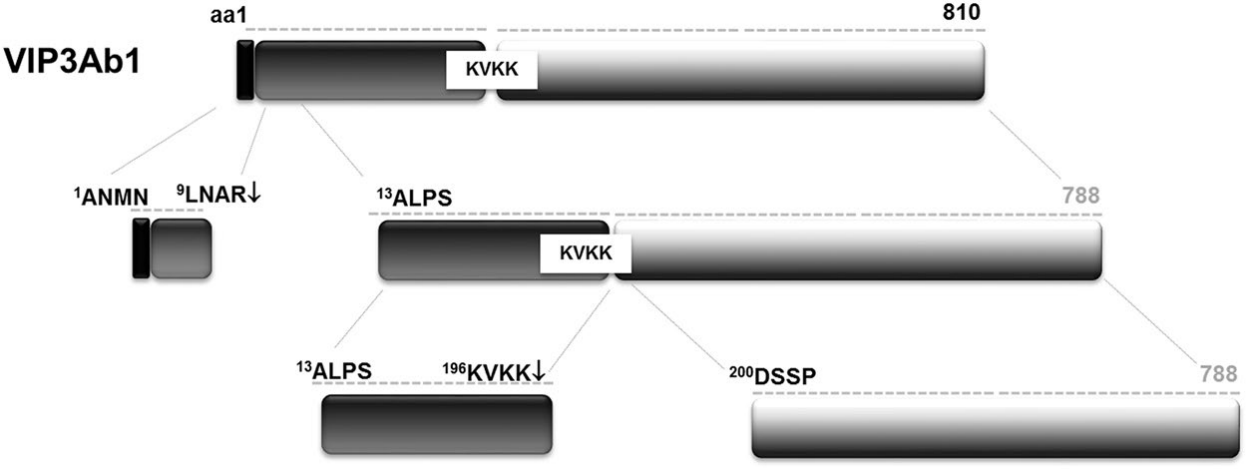
Stepwise processing of Vip3Ab1. For simplicity, numbering is based on the sequence numbering of full length Vip3Ab1. An initial cleavage site Arg^12^↓Ala^13^ removes the N-terminal 12 amino acids from Vip3Ab1. This is followed by a second processing site at Lys^199^↓Asp^200^ to produce ~20 kDa and 65 kDa fragments.

The second proteolytic step occurs in a region between amino acids ~200–220 of both Vip3 proteins in this study (see Fig. 8). Vip3Ab1 is cleaved after ^205^KVKK↓DSSP as expected, while Vip3Bc1 is processed at ^210^NVTK↓EVIE when digested with lepidopteran midgut enzymes. Motifs rich in basic amino acids such as arginine or lysine are often sites of serine protease recognition as in the case of proprotein convertases^26^. Therefore, processing after KVKK is anticipated considering the rich serine protease activity of the lepidopteran gut environment^27^. However, the Vip3Bc1 processing site was not predicted owing to the lack of an obvious cleavage motif. Vip3Bc1 rate of cleavage at ^210^NVTK↓EVIE is relatively slow compared to Vip3Ab1, which may be indicative of a less preferred yet exposed protease-sensitive site within the Vip3 structure. Additionally, this region may contain cleavage motifs favored by other proteinases.

### Vip3 N-terminal and C-terminal domains have specific interactions critical for proteolytic stability

Structural information for Cry1 and Cry2 Bt insecticidal proteins has helped define the mechanism of action and provided supporting explanation of differences in binding specificity. This information has been important towards evaluating the potential for cross resistance within the Cry1 family of insecticidal proteins. However, similar information has not yet been determined for Vip3 proteins. Kunthic *et al*. have overcome technical challenges to generate protein suitable for crystallization and have demonstrated Vip3A oligomerization before and after enzymatic processing^28^. More recent work has utilized transmission electron microscopy to reveal the surface topology of tetrameric Vip3Ag4 before and after processing^29^. Considering the oligomeric state and interaction between Vip3 domains, we sought to understand the importance of this association and function of these domains before and after processing by midgut enzymes in native conditions. We performed analytical SEC analysis to demonstrate that a member of the Vip3B family, Vip3Bc1, is also tetrameric in native conditions. Additionally, like Vip3Ab1, Vip3Bc1 persists as a tetramer after gut enzyme processing, which indicates that ~65 kDa and ~20 kDa products remain associated. Interaction between ~65 kDa and ~20 kDa has been demonstrated with Vip3Aa^30, 31^. However, this is the first demonstration the Vip3B proteins behave in a similar manner and suggests a common insecticidal mechanism within the Vip3 family. The precise mechanistic role of tetramer formation remains unclear. However, several observations provide insight towards domain function within Vip3 oligomers. First, we have observed the N-terminal portion of both Vip3Ab1 and Vip3Bc1 to be the most proteolytically stable region of the protein. Digestion experiments conducted with CEW and SBL gut enzymes in the presence of SDS show rapid (t_1/2_ < 1 minute) degradation of the ~65 kDa C-terminal region, while the N-terminal 20 kDa region is relatively stable (see Supplementary Fig. S1). Bel *et al*. (2017) observed that trypsinized full length Vip3Aa was readily degraded in denaturing conditions, but in native conditions the C-terminal domain remained stable^30^. However, we have expressed and purified the C-terminal domains (in absence of the N-terminal domain) of Vip3Ab1 and Vip3Bc1 and found them to be labile dimers prone to proteolysis as they are rapidly digested in native conditions. Thus, implicating the requirement for the N-terminal domain to maintain stability in proteolytic native environments. Lastly, in preparations of Vip3_BA chimeras containing tetrameric and monomeric species of Vip3_BA, Vip3 chimera monomers were rapidly degraded while tetrameric forms were stable for several hours. This indicates that tetramer formation may be able to stabilize Vip3 proteins in a proteolytic mileu. Also, despite a high degree of conservation within the N-terminal 20 kDa portion of Vip3 proteins, our data demonstrates that this domain is not interchangeable and suggests a specific association with the C-terminal region that provides stability and protects against complete proteolysis. Thus, the specific interaction between and amino and carboxy terminal domains appears to be important for oligomerization of Vip3 proteins, which further increases proteolytic stability in an alkaline proteolytic environment such as the lepidopteran midgut.

### The C-terminal ~65 kDa domain of Vip3 contributes specificity, but both the N-terminal and C-terminal domains are required for lethal activity

Functional evaluation of Vip3 proteins indicated a differential spectrum for Vip3Ab1 and Vip3Bc1. Vip3Bc1 was active on *O. nubilalis* and *P. includens*. Vip3Ab1 has a broader spectrum and is active on *H. zea*, *P. includens*, *S. frugiperda*, but not *O. nubilalis*. The Vip3_BA protein chimera, which contains the Vip3Ab1 C-terminal region, impaired the growth of the same pests as Vip3Ab1, indicating that the C-terminal portion of the protein drives specificity. This region of the Vip3A protein family spanning amino acids ~500–650 contains a predicted carbohydrate binding motif and is therefore thought to play a functional role in toxicity^5^. Additionally, a model of the C-terminal 200 amino acids of Vip3Aa indicates similarity to domain II of 3 domain Cry toxins, which suggests a role for the Vip3 C-terminal domain as a specificity determinant analogous to Cry toxins of known protein structure^32–34^. However, the lack of lethality indicates that the cognate N-terminal region is also important for full toxic effect. We hypothesize that this is due to increased susceptibility to proteolytic degradation in the midgut as we have observed *in vitro*. We observed a complete loss of lethality by both Vip3_AB and Vip3_BA chimeras, including on *P. includens*, an insect that was especially sensitive to Vip3Ab1 and Vip3Bc1. The loss of Vip3_AB activity was an unanticipated finding as this protein formed expected tetramers and was proteolytically stable, two attributes hypothesized to be required for lethal activity. Fang and colleagues^22^ generated more conservative Vip3A chimeric proteins within the C-terminal 179 amino acid region and noticed an increase in activity towards *O. nubilalis*, an insect previously non-susceptible to either parent Vip3A protein. However, this group reported an increase in growth inhibition and not lethality. Therefore, we conclude that the tetramers maintained via specific interactions of amino and carboxy terminal domains of Vip3 proteins not only provides proteolytic stability, but are also required to maintain the specificity determinants required for toxicity.

### Vip3Ab1 and Vip3Bc1 do not contain a toxic “core”

This study reveals a unique and necessary interaction between the N-terminal and C-terminal regions of two different members of the Vip3 insecticidal protein family. Therefore, our data do not support the existence of a “toxic core”. The majority of the extant literature has not directly inspected the activity of the C-terminal ~65 kDa fragment, as experimentation has been performed with full length Vip3A proteins that have been processed *in vitro* by trypsin^7, 8, 11, 19, 31, 35^ or isolated lepidopteran gut enzymes^12, 36, 37^. The insecticidal “core” has been inferred from the conserved nature of the N-terminal ~200 amino acids, the obvious presence of a large ~65 kDa fragment after processing, and the large collection of data on 3-domain Bt crystal toxins which demonstrate complete degradation of the C-terminal pro-domain in these proteins^38^. To our knowledge, two groups have expressed and purified the C-terminal portion of Vip3 from bacterial systems^39, 40^. Gayen *et al*. (2012) reported improvements in potency when the N-terminal 200 amino acids were deleted from the expressed Vip3A construct and later demonstrated that this truncated protein could confer protection against *Helicoverpa armigera, Agrotis ipsilon*, and *Spodoptera littoralis* in model plant systems. In contrast, others have observed complete loss of activity when the N-terminal 200 amino acids were omitted from another Vip3A expression construct^40^. They concluded that lack of activity was likely due to a chaperone-like function of the N-terminal ~200 amino acids required for proper folding and proteolytic stability of the C-terminal region. Our data aligns well with those of Li *et al*.^40^ as we report similar data with the ~65 kDa C-terminal region of Vip3Ab1 and Vip3Bc1. However, our work with Vip3 chimeras indicates that C-terminal portion of Vip3Bc1 is folded properly, forms tetramers, and is resistant to proteolysis when expressed as a chimera with the Vip3Ab1 N-terminus. However, the Vip3_AB chimera shows complete loss of activity against the four lepidopteran insects tested in this study; *H. zea*, *O. nubilalis*, *S. frugiperda* and *P. includens*. Thus, we propose that Vip3 proteins do not contain a toxic core similar to other Bt toxins, but rather require a proteolytic activation step that facilitates a change in conformation between ~20 kDa and ~65 kDa products that is important for stability and specificity.

In conclusion, the Vip family of Bt insecticidal proteins has become an attractive additional option to Bt crystal proteins for expression in crops to endow protection against a broad spectrum of lepidopteran pests. This is evidenced by the commercial success of transgenic Viptera™ corn and VipCot™cotton, which have both been engineered to produce Vip3Aa proteins. Despite the demonstrated agronomic value, little work has been done to understand the commercial potential and biochemical nature of the Vip3 protein family. In this work, we characterized 2 novel insecticidal proteins; Vip3Ab1 and Vip3Bc1. Through this work, we have 1) uncovered a critical role in maintaining protein stability for a the highly conserved N-terminal region, 2) validated the C-terminal region as the major determinant of specificity, and 3) demonstrated a specific interaction between N-terminal and C-terminal regions that permits the formation of a lethal toxin. We believe these studies will serve as a foundation towards a more thorough understanding of the Vip3 family of proteins.

## Materials and Methods

### Design and build of native and chimeric Vip3 proteins

Vip3 genes used in this work,*vip3Ab1* (GenBank accession AAR40284) and *vip3Bc1* (GenBank accession MF543028), were codon biased for expression in maize. These 2 genes were synthesized and used as templates for amplification. PCR amplification was completed using the Platinum Taq DNA Polymerase (Thermo Fisher, Waltham, MA) and the manufacturer’s protocol. Primers were designed to amplify parts from Vip3Ab1 and Vip3Bc1 to create chimeras by homologous recombination (see Supplementary Table S2). PCR amplicons were cleaned using the QIAquick PCR Purification Kit (Qiagen, Hilden, Germany) and the manufacturer’s protocol. Construction strategy for the parts is shown in Supplementary Table S3. Parts were combined into an *E. coli* backbone for verification. The chimeras were each sequence verified using Sanger sequencing (Eurofins Genomics, Louisville, KY). Once verified the chimeras were digested from the *E. coli* backbone using restriction enzymes SpeI and SalI (New England Biolabs, Ipswich, MA) to be ligated into a compatibly digested *Pseudomonas flourescens* (*Pf*) expression vector. Ligation was performed using T4 DNA Ligase (New England Biolabs, Ipswich, MA) following the manufacturer’s protocol with 50ng of the digested GOI and 25ng of the *Pf* expression vector. The ligation mixture was transformed into *Pf* competent cells and the final constructs were validated via multiple restriction digestions.

### Protein expression and purification

All Vip proteins were expressed in recombinant *Pseudomonas fluorescens* strains as described previously^41^. Expression of *vip* genes from the *Ptac* promoter was induced by addition of isopropyl-β-D-1-thiogalactopyranoside (IPTG) after an initial incubation of 24 hours at 30 °C with shaking in M9 medium supplemented with 1% glucose, trace elements and 5% glycerol. Harvested *P. fluorescens* cells were sonicated in lysis buffer consisting of 50 mM sodium phosphate (pH 8.0), 5% glycerol, 5 mM benzamidine HCl, 5 mM TCEP and 2 mM EDTA. The extract was centrifuged at 20,000 × *g* for 60 minutes. The soluble protein in the supernatant was precipitated with 50% ammonium sulfate and centrifuged at 20,000 × *g* for 20 minutes. The pellet was resuspended in 50 mM sodium phosphate (pH 8.0) and purified by anion exchange chromatography using a HiTrap™ Q HP 5 mL column with an AKTA Purifier chromatography system (GE Healthcare, UK). The column was equilibrated in 50 mM sodium phosphate(pH 8.0), and proteins were eluted with a stepwise gradient to 1 M NaCl. Protein-containing fractions were combined and concentrated using Amicon Ultra-15 Centrifugal Filter Devices with a 30 kDa MWCO (EMD Millipore). Proteins were desalted to 50 mM sodium phosphate (pH 8.0) for bioassay using Zeba® Spin Desalting Columns, 7 MWCO (Thermo Scientific, Waltham, MA). Total protein concentrations were measured with the NanoDrop 2000C Spectrophotometer (Thermo Scientific, Waltham, MA), using the A280 method.

### SDS-PAGE analysis and N-terminal amino acid sequencing

SDS-PAGE analysis was performed using NuPAGE® Novex 4–12% Bis-Tris Protein Gels (Thermo Scientific, Waltham, MA). Proteins were diluted 4X in NuPAGE® LDS Sample Buffer (Thermo Scientific, Waltham, MA) containing 100 mM TCEP prior to loading onto the gel. Ten µL of Novex Sharp Pre-stained Protein Standard was loaded onto one lane of each gel. Gels were run according to the manufacturer’s recommendations using NuPAGE® MES SDS Running Buffer and stained with SimplyBlue SafeStain, then destained in water and imaged on a flatbed scanner.

Samples were prepared for N-terminal sequencing by blotting an SDS-PAGE gel onto a PVDF pre-cut blotting membrane (Thermo Scientific, Waltham, MA) via wet tank transfer in 10 mM CAPS (pH 11) with 10% methanol. The PVDF membrane was stained for 15–20 seconds with Coomassie Brilliant Blue (Bio-Rad, Hercules, CA), destained in 45% methanol; 10% acetic acid, rinsed with water and air dried. The target bands were excised and analyzed by Edman degradation using a Shimadzu PPSQ-33A protein sequencer (Shimadzu, Kyoto, Japan). The data was analyzed with Shimadzu data analysis software.

### Midgut juice Vip protein digestion

Midguts were dissected from insects and placed into vials containing 8.5% sucrose and 150 mM NaCl. Soluble proteins, including digestive proteases, were isolated by centrifugation for 30 minutes at 10,000 x g. Proteolytic activity was normalized using BODIPY-casein degradation assay. In this assay, fluorescein labeled casein is incubated at 10 µg/mL 50 mM 3-(N-morpholino)propanesulfonic acid (MOPS) with extract and fluorescence is monitored over time as relative fluorescence units per second (RFU). Vip proteins were digested with Corn Earworm (*Helicoverpa zea*) or Soybean Looper (*Pseudoplusia includens*) gut fluid at 4 µL/mL and 8 µL/mL, respectively. Proteins were added to the reaction for a final concentration of 150 µg/mL in 50 mM MOPS pH 10 buffer. Control reactions were prepared containing no insect gut fluid, except for size exclusion controls, which contained gut fluids inactivated by heating at 95 °C for 15 minutes. Reactions were incubated at 30 °C with shaking for varying time intervals. Protease Inhibitor Cocktail (Sigma-Aldrich, St. Louis, MO) was added to terminate the reactions prior to SDS-PAGE and size exclusion analysis.

### Analytical size

#### exclusion chromatography

All analyses were performed on an Agilent 1200 HPLC (Agilent, Santa Clara, CA) using a TSKgel SuperSW3000 2.0 mm × 30 cm, 4 µm column (Tosoh Biosciences Tokyo, Japan) isocratically in 50 mM sodium phosphate pH 7.0 containing 250 mM NaCl at a flow rate 75 µl/min for 30 min. Injection volumes were 3 µl at a protein concentration of 150 µg/ml. Protein molecular weight standards were purchased from Sigma (#69385) and reconstituted according to the manufacturer’s directions. Fluorescence detection was monitored at 295 nm excitation and 345 nm emission. Data analysis was performed using ChemStation software (Agilent).

### Insect Bioassays

Proteins were tested for insecticidal activity using neonate Lepidopteran larvae on artificial insect diet. Larvae of *H. zea*, *O. nubilalis*, *S. frugiperda* and *P. includens* were hatched from eggs obtained from Benzon Research Inc. (Carlisle, PA). The bioassays were conducted in 128-well plastic trays specifically designed for insect bioassays (C-D International, Pitman, NJ). Each well contained 1.5 mL of Multi-species Lepidoptera diet (Southland Products, Lake Village, AR). A 40 µL aliquot of protein sample was delivered by pipette onto the 2 cm^2^ diet surface of each well (20 μL/cm^2^). Treatment concentrations were calculated as the amount (ng) of protein per square centimeter (cm^2^) of surface area in the well. The treated trays were held in a fume hood until the liquid on the diet surface had evaporated or was absorbed into the diet. Within a few hours of eclosion, individual larvae were picked up with a moistened camel hair brush and deposited on the treated diet, one larva per well. Sixteen animals were used per treatment. The infested wells were then sealed with adhesive sheets of clear plastic, vented to allow gas exchange (C-D International, Pitman, NJ). Bioassay trays were held at 28 °C, ~40% relative humidity and 16:8 hours light:dark for 5 days, after which the total number of insects exposed to each protein sample, the number of dead insects and number of moribund insects were recorded. Moribund insects were classified as insects that were still alive, did not increase in size over the course of the bioassay, and did not respond to perturbation.

Statistical analysis was carried out using JMP® Pro Version 11 software (SAS Institute Inc., Cary, NC). Lethal concentrations (LC_50_) of Vip3 proteins were calculated on sum of dead and moribund insects using a generalized linear model utilizing Probit analysis of binomial data. An inverse prediction of LC50 with 95% confidence intervals was calculated based on this model. For determination of growth inhibition, total live insect mass was weighed after 5 days of treatment and normalized for insect number. Sixteen insects were used per treatment and experiments were repeated twice. Insects tested on buffer control diet were compared to Vip3-treated samples. Average weight of insects after Vip3 treatment was modeled using linear mixed models for each insect species respectively:

$$Y=\mu +T+E+\varepsilon $$

where Y is the insect average weight observed in each treated well, µ is the overall mean, T is the effect of Vip3 protein at a specific dose, E is the effect of different experimental run, ɛ is the error. The Vip3 protein treatment effect T was modeled as a fixed effect, while experimental run E was treated as a random effect. Box-Cox transformation was applied to correct for non-normality and heterogeneous variances. Average insect weight was compared with null hypothesis of no growth difference between a Vip3 protein treatment and the untreated control (buffer) and the alternative hypothesis of different growth under a Vip3 protein treatment. We used Dunnett’s test to adjust P-values for multiple comparisons.

All data generated and analysed during this study are included in this published article.

## Electronic supplementary material

Supplementary File

## References

[1] Jurat-Fuentes, J. L. & Crickmore, N. Specificity determinants for Cry insecticidal proteins: Insights from their mode of action. J Invertebr Pathol, doi:10.1016/j.jip.2016.07.018 (2016). 10.1016/j.jip.2016.07.018 27480404

[2] Pardo-Lopez L, Soberon M, Bravo A (2013). Bacillus thuringiensis insecticidal three-domain Cry toxins: mode of action, insect resistance and consequences for crop protection. FEMS Microbiol Rev.

[3] Estruch JJ (1996). Vip3A, a novel Bacillus thuringiensis vegetative insecticidal protein with a wide spectrum of activities against lepidopteran insects. Proc Natl Acad Sci USA.

[4] Bi Y (2015). Genomic sequencing identifies novel Bacillus thuringiensis Vip1/Vip2 binary and Cry8 toxins that have high toxicity to Scarabaeoidea larvae. Appl Microbiol Biotechnol.

[5] Chakroun M, Banyuls N, Bel Y, Escriche B, Ferre J (2016). Bacterial Vegetative Insecticidal Proteins (Vip) from Entomopathogenic Bacteria. Microbiol Mol Biol Rev.

[6] Yu X (2011). Rapid detection of vip1-type genes from Bacillus cereus and characterization of a novel vip binary toxin gene. FEMS Microbiol Lett.

[7] Chakroun M (2012). Susceptibility of Spodoptera frugiperda and S. exigua to Bacillus thuringiensis Vip3Aa insecticidal protein. J Invertebr Pathol.

[8] Hernandez-Martinez P, Hernandez-Rodriguez CS, Rie JV, Escriche B, Ferre J (2013). Insecticidal activity of Vip3Aa, Vip3Ad, Vip3Ae, and Vip3Af from Bacillus thuringiensis against lepidopteran corn pests. J Invertebr Pathol.

[9] Song F (2016). Insecticidal activity and histopathological effects of Vip3Aa protein from Bacillus thuringiensis on Spodoptera litura. J Microbiol Biotechnol.

[10] Current and Previously Registered Section 3 Plant-Incorporated Protectant (PIP) Registrations. Environmental Protection Agency Website (2016).

[11] Lee MK, Miles P, Chen JS (2006). Brush border membrane binding properties of Bacillus thuringiensis Vip3A toxin to Heliothis virescens and Helicoverpa zea midguts. Biochem Biophys Res Commun.

[12] Lee MK, Walters FS, Hart H, Palekar N, Chen JS (2003). The mode of action of the Bacillus thuringiensis vegetative insecticidal protein Vip3A differs from that of Cry1Ab delta-endotoxin. Appl Environ Microbiol.

[13] Liu JG, Yang AZ, Shen XH, Hua BG, Shi GL (2011). Specific binding of activated Vip3Aa10 to Helicoverpa armigera brush border membrane vesicles results in pore formation. J Invertebr Pathol.

[14] Yu CG, Mullins MA, Warren GW, Koziel MG, Estruch JJ (1997). The Bacillus thuringiensis vegetative insecticidal protein Vip3A lyses midgut epithelium cells of susceptible insects. Appl Environ Microbiol.

[15] Crickmore, N. *et al*. Bacillus thuringiensis toxin nomenclature, http://www.btnomenclature.info (2016).

[16] Crickmore N (1998). Revision of the nomenclature for the Bacillus thuringiensis pesticidal crystal proteins. Microbiol Mol Biol Rev.

[17] de Maagd RA, Bravo A, Berry C, Crickmore N, Schnepf HE (2003). Structure, diversity, and evolution of protein toxins from spore-forming entomopathogenic bacteria. Annu Rev Genet.

[18] Rang C, Gil P, Neisner N, Van Rie J, Frutos R (2005). Novel Vip3-related protein from Bacillus thuringiensis. Appl Environ Microbiol.

[19] Ben Hamadou-Charfi D, Boukedi H, Abdelkefi-Mesrati L, Tounsi S, Jaoua S (2013). Agrotis segetum midgut putative receptor of Bacillus thuringiensis vegetative insecticidal protein Vip3Aa16 differs from that of Cry1Ac toxin. Journal of Invertebrate Pathology.

[20] Gouffon C, Van Vliet A, Van Rie J, Jansens S, Jurat-Fuentes JL (2011). Binding Sites for Bacillus thuringiensis Cry2Ae Toxin on Heliothine Brush Border Membrane Vesicles Are Not Shared with Cry1A, Cry1F, or Vip3A Toxin. Appl Environ Microb.

[21] Sena JA, Hernandez-Rodriguez CS, Ferre J (2009). Interaction of Bacillus thuringiensis Cry1 and Vip3A proteins with Spodoptera frugiperda midgut binding sites. Appl Environ Microbiol.

[22] Fang, J. *et al*. Characterization of chimeric Bacillus thuringiensis Vip3 toxins. Appl Environ Microbiol 73, 956–961, doi:AEM.02079-06 (2007) 10.1128/AEM.02079-06 PMC1800787 17122403

[23] Chen J (2003). Comparison of the expression of Bacillus thuringiensis full-length and N-terminally truncated vip3A gene in Escherichia coli. J Appl Microbiol.

[24] Selvapandiyan A (2001). Toxicity analysis of N- and C-terminus-deleted vegetative insecticidal protein from Bacillus thuringiensis. Appl Environ Microbiol.

[25] Sellami S, Cherif M, Jamoussi K (2016). Effect of adding amino acids residues in N- and C-terminus of Vip3Aa16 (L121I) toxin. J Basic Microbiol.

[26] Seidah NG, Chretien M (1999). Proprotein and prohormone convertases: a family of subtilases generating diverse bioactive polypeptides. Brain Res.

[27] Purcell JP, Greenplate JT, Sammons RD (1992). Examination of Midgut Luminal Proteinase Activities in 6 Economically Important Insects. Insect Biochem Molec.

[28] Kunthic T, Surya W, Promdonkoy B, Torres J, Boonserm P (2016). Conditions for homogeneous preparation of stable monomeric and oligomeric forms of activated Vip3A toxin from Bacillus thuringiensis. Eur Biophys J.

[29] Palma, L. *et al*. The Vip3Ag4 Insecticidal Protoxin from Bacillus thuringiensis Adopts A Tetrameric Configuration That Is Maintained on Proteolysis. *Toxins (Basel)* **9**, doi:10.3390/toxins9050165 (2017). 10.3390/toxins9050165 PMC5450713 28505109

[30] Bel, Y., Banyuls, N., Chakroun, M., Escriche, B. & Ferre, J. Insights into the Structure of the Vip3Aa Insecticidal Protein by Protease Digestion Analysis. *Toxins (Basel)* **9**, doi:10.3390/toxins9040131 (2017). 10.3390/toxins9040131 PMC5408205 28387713

[31] Chakroun M, Ferre J (2014). *In vivo* and *in vitro* binding of Vip3Aa to Spodoptera frugiperda midgut and characterization of binding sites by (125)I radiolabeling. Appl Environ Microbiol.

[32] Schnepf E (1998). Bacillus thuringiensis and its pesticidal crystal proteins. Microbiol Mol Biol Rev.

[33] Wu J (2007). Evidence for positive Darwinian selection of Vip gene in Bacillus thuringiensis. J Genet Genomics.

[34] Xu C, Wang BC, Yu Z, Sun M (2014). Structural insights into Bacillus thuringiensis Cry, Cyt and parasporin toxins. Toxins (Basel).

[35] Caccia S, Chakroun M, Vinokurov K, Ferre J (2014). Proteolytic processing of Bacillus thuringiensis Vip3A proteins by two Spodoptera species. J Insect Physiol.

[36] Abdelkefi-Mesrati L (2011). Investigation of the steps involved in the difference of susceptibility of Ephestia kuehniella and Spodoptera littoralis to the Bacillus thuringiensis Vip3Aa16 toxin. J Invertebr Pathol.

[37] Abdelkefi-Mesrati L (2011). Study of the Bacillus thuringiensis Vip3Aa16 histopathological effects and determination of its putative binding proteins in the midgut of Spodoptera littoralis. J Invertebr Pathol.

[38] de Maagd RA, Bravo A, Crickmore N (2001). How Bacillus thuringiensis has evolved specific toxins to colonize the insect world. Trends Genet.

[39] Gayen S, Hossain MA, Sen SK (2012). Identification of the bioactive core component of the insecticidal Vip3A toxin peptide of Bacillus thuringiensis. J Plant Biochem Biot.

[40] Li C (2007). Bacillus thuringiensis Vip3 mutant proteins: Insecticidal activity and trypsin sensitivity. Biocontrol Sci Techn.

[41] Squires C. H., R. D., Chew L. C., Ramseier T. M., Schneider J. C., Talbot W. Heterologous protein production in P. fluorescens. BioProcess International, 54–58 (2004).

