# Supplementary material for: Functional characterization of Vip3Ab1 and Vip3Bc1: Two novel insecticidal proteins with differential activity against lepidopteran pests
Source: Sci Rep. 2017 Sep 11;7:11112. doi: 10.1038/s41598-017-11702-2 (PMC5593919; doi:10.1038/s41598-017-11702-2)
Supplement: Supplementary file 1 — Supplementary File [file 41598_2017_11702_MOESM1_ESM.pdf]

Functional characterization of Vip3Ab1 and Vip3Bc1: Two novel insecticidal proteins with differential activity against lepidopteran pests

Marc D. Zack*[1], Megan S. Sopko[1], Meghan L. Frey[1], Xiujuan Wang[1], Sek Yee Tan[1], Jennifer M. Arruda[1], Ted T. Letherer[1], & Kenneth E. Narva[1]

* Corresponding Author

[1] Dow AgroSciences, 9330 Zionsville Road

Indianapolis, IN 46268

Email:mdzack@dow.com

Supplementary Data.

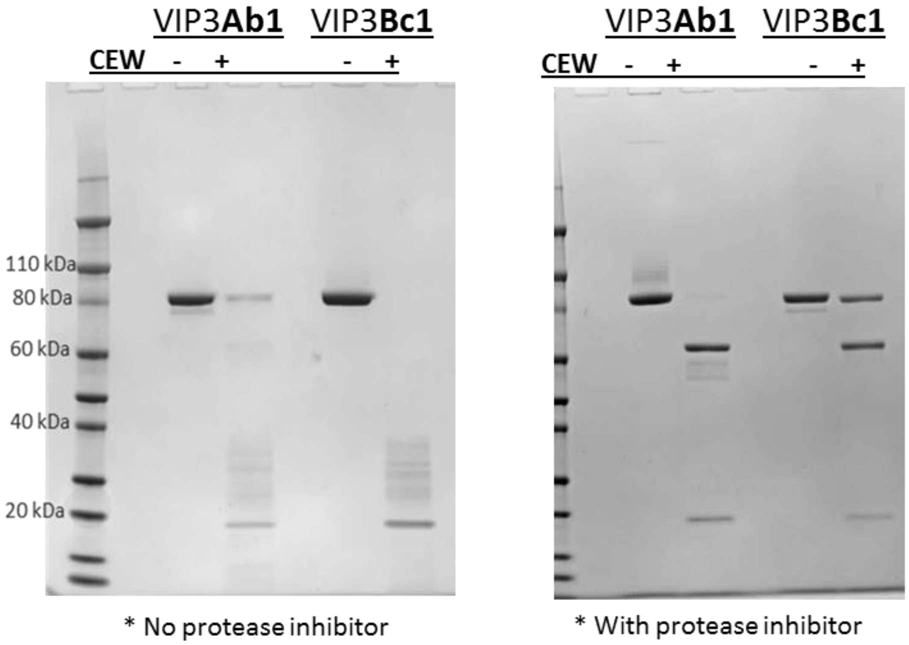

Figure S1. Digestion of Vip3Ab1 and Vip3Bc1 with CEW gut enzymes. Left – Digestion of Vip3 proteins overnight at 30°C and analyzed by LDS-PAGE in absence of protease inhibitor shows complete degradation of ~65 kDa carboxy terminal fragments and presence of ~20 kDa amino terminal fragments. Right – digestions were stopped with the addition of protease inhibitors and allowed to sit overnight at 4°C before SDS-PAGE analysis. In this case, both the ~65 kDa and ~20 kDa products are apparent. N-terminal sequencing was performed on the ~20 kDa band and indicated an N-terminus of ALPSF for both proteins (Alanine #21 and #13 for Vip3Bc1 and Vip3Ab1, respectively). Results indicates stability of the ~20 kDa fragment in the presence of denaturing conditions where the ~65 kDa fragment is readily digested.

Primer Sequences

Primer Name	Primer Sequence
Vip3AbN_For	TTAATCTACTAGTAGGAGGTAACTTATGGCAAACATGAACAACACCAAAC
Vip3AbN_Rev	**CTCAGTCAATTCATCAAGGATATCG**
Vip3BcC_For	**CGATATCCTTGATGAATTGACTGAG**CTTACTGAGCTGACGGAGCTGG
Vip3BcC_Rev	CGAAAGGCCCAGTCTGTCGACTCACTCCTTCACGATGCTCACGTTGGAG
Vip3BcN_For	TTAATCTACTAGTAGGAGGTAACTTATGGTGCAGAAGTGGATGCAGAGG
Vip3BcN_Rev	**CTCGTTCAGGTTCTCGATCACCTCC**
Vip3AbC_For	**GGAGGTGATCGAGAACCTGAACGAG**CTTGCAAAGTCCGTGACCAAGAATG
Vip3AbC_Rev	CGAAAGGCCCAGTCTGTCGACCTACTTGATCGAGAAATCGCGAAAGTTGATGG

Table S2. Primer parts used in construction of Vip3 chimeras. Grey regions indicate homology regions to E. coli backbone. Bold indicates homology between parts.

	Part A		Part B	
Chimera	Fprimer	Rprimer	Fprimer	Rprimer
Vip3Ab1 (Met1-Glu212) + Vip3Bc1 (Leu222-Glu803)	3AbN_F	3AbNHto3BcCA	3AbNAto3BcCH	3BcC_R
Vip3Bc1 (Met1-Glu221) + Vip3Ab1 (Leu213-Lys788)	3BcN_F	3BcNHto3AbCA	3BcNAto3AbCH	3AbC_R

Table S3. Chimera construction strategy
